# Melatonin synergizes BRAF‐targeting agent dabrafenib for the treatment of anaplastic thyroid cancer by inhibiting AKT/hTERT signalling

**DOI:** 10.1111/jcmm.15854

**Published:** 2020-09-15

**Authors:** Yina Liao, Yao Gao, An Chang, Zongjuan Li, Huayu Wang, Jing Cao, Wei Gu, Ranran Tang

**Affiliations:** ^1^ Shanghai Center for Thyroid Disease Shanghai Tenth People's Hospital School of Medicine Tongji University Shanghai China; ^2^ Department of Endocrinology Children's Hospital of Nanjing Medical University Nanjing China; ^3^ Department of Drug Administration First affiliated Hospital of Jinzhou Medical University Jinzhou China; ^4^ The Second Affiliated Hospital and Institute of Cancer Stem Cell Dalian Medical University Dalian China; ^5^ Nanjing Maternity and Child Health Care Hospital Women’s Hospital of Nanjing Medical University Nanjing China

**Keywords:** AKT/hTERT, anaplastic thyroid cancer, dabrafenib, melatonin

## Abstract

As a selective inhibitor of BRAF kinase, dabrafenib has shown potent anti‐tumour activities in patients with BRAFV600E mutant anaplastic thyroid cancer. However, the resistance of thyroid cancer cells to dabrafenib limited its therapeutic effect. The effects of melatonin and dabrafenib as monotherapy or in combination on the proliferation, cell cycle arrest, apoptosis, migration and invasion of anaplastic thyroid cancer cells were examined. The molecular mechanism involved in drug combinations was also revealed. Melatonin enhanced dabrafenib‐mediated inhibition of cell proliferation, migration and invasion, and promoted dabrafenib‐induced apoptosis and cell cycle arrest in anaplastic thyroid cancer cells. Molecular mechanistic studies further uncovered that melatonin synergized with dabrafenib to inhibit AKT and EMT signalling pathways. Furthermore, melatonin and dabrafenib synergistically inhibited the expression of hTERT, and the inhibition of cell viability and the induction of cell cycle arrest mediated by the combination of these two drugs were reversed by hTERT overexpression. Taken together, our results demonstrated that melatonin synergized the anti‐tumour effect of dabrafenib in human anaplastic thyroid cancer cells by inhibiting multiple signalling pathways, and provided new insights in exploring the potential therapeutic targets for the treatment of anaplastic thyroid cancer.

## 1 INTRODUCTION

Thyroid cancer has become one of the utmost frequent malignancies of the endocrine system, and their incidence has been increasing rapidly worldwide in recent decades.^1^ In 2018, there were approximately 40 900 newly diagnosed thyroid cancer cases among women in the United States,^2^ accounting for 5.1% of the total estimated cancer burden in women.^3^ According to cell origin and histological characteristics, thyroid cancers are usually divided into follicular thyroid cancer (FTC), papillary thyroid cancer (PTC), medullary thyroid cancer (MTC) and anaplastic thyroid cancer (ATC). Among them, FTC, PTC and ATC are derived from the follicular thyrocytes, while MTC is the only parafollicular C‐cell‐derived tumour. Approximately 90% of thyroid cancers are PTC and FTC, which are usually curable with a 10‐year survival rate of more than 85%.^4^ In addition, about 3% of thyroid cancer cases are MTC, and the overall 5‐year survival rate is over 80%.^5^ ATC is rare, accounting for only 1% to 2% of all thyroid cancers, but leads to the majority of thyroid cancer‐related deaths, making it one of the most aggressive human malignancies.^6^, ^7^ Patients diagnosed with ATCs have a median survival of 3‐6 months due to its aggressive growth, extrathyroidal invasion, distant metastasis and resistance to conventional treatment.^8^, ^9^ To date, there is no standard or effective therapy to prolong the survival of ATC patients.^7^, ^10^, ^11^ Therefore, there is an urgent need to identify new biological targets that can be converted into therapeutic approaches.

Recently, several driver mutations have been identified in ATC, resulting in a better understanding of the development of this heterogeneous disease.^12^ BRAF mutation, specifically BRAFV600E mutation, occurs in approximately 25% of ATC.^13^ This mutation leads to constitutive activation of the BRAF kinase, followed by phosphorylation of MEK and ERK to induce oncogenic activation of the mitogen‐activated protein kinase (MAPK) pathway which transmits mitogenic signals from the cell membrane to the nucleus and plays a critical role in mediating cellular proliferation, differentiation, apoptosis and survival.^7^, ^14^, ^15^ Dabrafenib, an effective ATP competitive inhibitor which selectively inhibits the activity of BRAFV600E kinase, displays impressive initial response rates.^16^, ^17^ Unfortunately, the perdurability of response is limited owing to the drug resistance.^17^, ^18^ Therefore, overcoming the acquired resistance to BRAFV600E mutation inhibitors and understanding the underlying mechanisms are critical for improving patient outcomes.

Melatonin (N‐acetyl‐5‐methoxytryptamine) is an indoleamine compound, most of which is synthesized and secreted by the pineal gland. It is well known that melatonin is involved in the regulation of circadian rhythms and endocrine functions.^19^ Actually, melatonin has been reported to participate in many other functions, including antioxidation, anti‐angiogenesis, anti‐inflammatory and activation of the immune system.^20^, ^21^, ^22^, ^23^ In recent years, increasing evidence has demonstrated that melatonin has anti‐tumour effect in various cancers, such as melanoma, breast cancer, lung carcinoma and thyroid cancer.^24^, ^25^, ^26^, ^27^ Melatonin has been shown to be an effective combination therapy for cancers by improving the curative effect of conventional anticancer drugs, reducing their side effects and overcoming their resistance to chemotherapy.^28^, ^29^ However, the combination of melatonin and BRAF‐targeting agent dabrafenib for the treatment of anaplastic thyroid cancer has not been reported.

In this study, we evaluated the role of melatonin in enhancing dabrafenib‐mediated anti‐tumour effects and uncovered the potential molecular mechanisms of this combination therapy in anaplastic thyroid cancer.

## 2 MATERIALS AND METHODS

### 2.1 Cell lines and culture conditions

Human anaplastic thyroid cancer cell lines SW1736, OCUT1, KHM‐5M and CAL‐62 were all obtained from the American Type Culture Collection (ATCC). The cells were maintained in Dulbecco's modified Eagle's medium (DMEM), which contained 10% foetal bovine serum and antibiotics (100 U/mL penicillin and 100 g/mL streptomycin).

### 2.2 Reagents and antibodies

Melatonin (#A2842) and dabrafenib (#B1407) were purchased from Apexbio (USA). The antibodies against β‐actin (#20536‐1‐AP), E‐cadherin (#20874‐1‐AP), N‐cadherin (#22018‐1‐AP), vimentin (#10366‐1‐AP), cyclin D1 (#60186‐1‐Ig) and CDK2 (#10122‐1‐AP) were purchased from Proteintech (USA). Antibodies against Bcl‐2 (#4223), Bax (#5023), cleaved PARP (#5625), p‐AKT (#4060), AKT (#4691), cleaved caspase‐3 (#9664) and caspase‐3 (#14220) were purchased from Cell Signaling Technology (USA). The anti‐hTERT antibody (#ab32020) was purchased from Abcam (USA), and anti‐MMP‐9 antibody (#AF5228) was purchased from Affinity Biosciences (USA).

### 2.3 Western blot

Cells were lysed on ice in protein extraction reagent, and protein concentration was determined by using BCA Protein Assay Kit (Thermo Fisher Scientific, USA). Equal amounts of proteins were loaded onto 10%‐15% gradient SDS‐PAGE gels and then transferred onto polyvinylidene fluoride membranes. Western blots were immunoblotted with the specific primary antibodies, followed by incubation with HRP‐conjugated secondary antibody, finally detected by enhanced chemiluminescence.

### 2.4 Cell viability assay

Thyroid cancer cells were seeded into 96‐well plates (4000‐6000 cells/well) and cultured with different treatment. Cell viability was assessed by the CCK‐8 assay (Apexbio, USA), and the absorbance was measured at the wavelength of 450 nm.

Synergistic effect assessment of melatonin and dabrafenib was based on cell viability. The combination index (CI) values, using Chou and Talalay methods, have been widely used to characterize drug interactions. CI ˂1, =1 and ˃1 represented synergism, additive and antagonism effects, respectively.

### 2.5 Colony formation assay

Thyroid cancer cells treated with the indicated doses of melatonin or dabrafenib were harvested and counted. Approximately 1000 cells were seeded in triplicate into 6‐well plates and incubated for 8‐14 days until grew into macroscopic colonies. Then, the cells were washed with PBS solution, fixed for 15 minutes and stained with 0.1% crystal violet for 20 minutes. The clones that contained more than 50 cells were counted and photographed.

### 2.6 Scratch assay

Scratch assay was used to detect cell migration ability. SW1736 and OCUT1 cells were plated in 6‐well plates and grown to a confluence of approximately 70%. The cells were then scraped in a straight line to create a ‘scratch’ and treated with melatonin or dabrafenib, alone or in combination. The wound gap was photographed by inverted microscope at 0 and 48 hours.

### 2.7 Transwell invasion assay

1 × 10^4^ thyroid cancer cells with indicated treatment were suspended in 100 μL serum‐free medium and added to each matrigel‐coated upper chamber (Corning, USA), while the lower chambers were filled with 500 μL medium containing 20% foetal bovine serum. After incubation at 37°C for 24 hours, the cells that did not invade through the pores were removed with a cotton swab. The chambers were washed, fixed, stained with 0.1% crystal violet and counted in three random view fields.

### 2.8 Cell cycle assay

Thyroid cancer cells were collected after treatment with melatonin or dabrafenib, and cell cycle was analysed using a Cell Cycle Detection Kit (KeyGen Biotech, China). Cells were sorted by FACSCanto II Flow Cytometer (BD Biosciences, USA), and the relative proportions of cells in the G1, S and G2‐M phases of the cell cycle were analysed by using FlowJo 7.6 software.

### 2.9 Cell apoptosis assay

Detection of cell apoptosis was based on FACS analysis by FITC‐AV/PI staining. Cells were plated in 6‐well plates and treated with the indicated concentration of melatonin or dabrafenib. Cell apoptosis assay was performed using an Annexin V‐FITC Apoptosis Detection Kit (KeyGen Biotech, China). The status of cell apoptosis was analysed by FACSCanto II Flow Cytometer (BD Biosciences, USA).

### 2.10 RNA extraction and real‐time qPCR

Total RNA was extracted from thyroid cancer cells by using TRIzol reagent (Life Technologies, USA) according to the manufacturer's instructions. cDNA was synthesized by using TransScript One‐Step gDNA Removal and cDNA Synthesis SuperMix (TRAN). To assess mRNA expression, qRT‐PCR was conducted using SYBR Premix Ex Taq™ II (TaKaRa, Japan). The target gene expression was normalized to the expression of GAPDH and analysed with the 2^−△△Ct^ methods. The primer sequences used were hTERT (forward 5'‐CCCATTTCATCAGCAAGTTTGG −3' and reverse 5'‐CTTGGCTTTCAGGATGGAGTAG‐3') and GAPDH (forward 5'‐TGT GGGCATCAATGGATTTGG‐3' and reverse 5'‐ACACCATGTATTCCGGGTCAAT‐ 3').

### 2.11 Statistical analysis

All experiments were performed three times, and the results were shown as mean ± SD. To compare the statistical differences, GraphPad Prism software was used by two‐tailed Student's *t* test or one‐way ANOVA as approximate. *P* value less than 0.05 was considered significant.

## 3 RESULTS

### 3.1 Melatonin and dabrafenib synergized to inhibit the proliferation of anaplastic thyroid cancer cells

**FIGURE 1:**
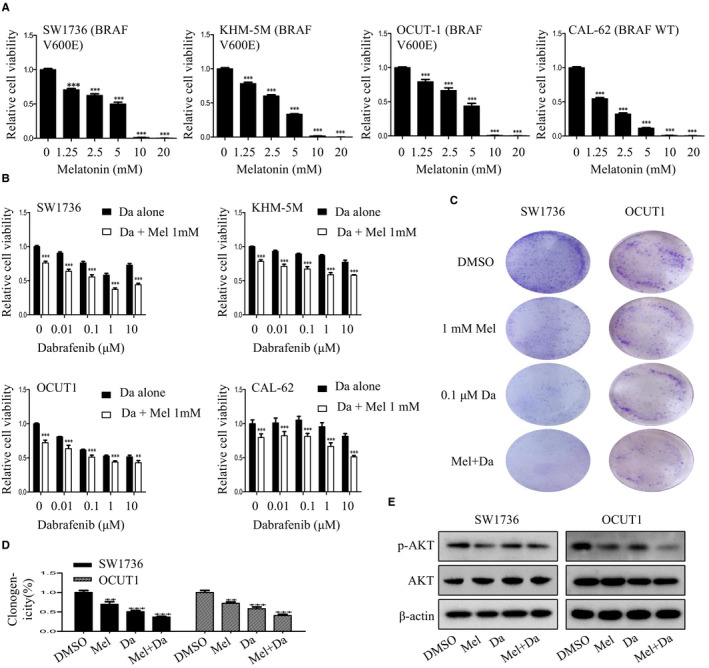
Melatonin enhanced dabrafenib‐mediated cell proliferation inhibition. A, Human anaplastic thyroid cancer cells were treated with melatonin at the indicated doses. After 48 h, cell viability was determined by CCK‐8 assay. B, Human anaplastic thyroid cancer cells were treated with the indicated doses of dabrafenib (Da) alone or combined with melatonin (Mel) (1mM) for 48 h, and cell viability was examined by CCK‐8 assay. C and D, SW1736 and OCUT1 cells were treated with Mel (1 mmol/L) or Da (0.1 μmol/L) alone or their combination. The colony formation results were photographed (C), and their relative numbers were counted (D). E, The expression of AKT signalling–related proteins p‐AKT and AKT in anaplastic thyroid cancer cells with indicated treatment was respectively detected by Western blot assays. Data were presented as mean ± SD of three independent experiments. The level of significance was indicated by ***P < .001, **P < .01

To study the role of melatonin in dabrafenib‐mediated cell proliferation inhibition, we first examined the cell viability of melatonin and dabrafenib as single agents or in combination in a panel of human ATC cell lines. The CCK‐8 assay showed that melatonin alone, at the dose of 1.25‐20 mmol/L, obviously inhibited cell viability in a dose‐dependent manner (Figure 1A). Dabrafenib alone, at the dose from 0.01 to 10 μmol/L, also inhibited the viability of BRAFV600E mutant cells in a concentration‐dependent manner, while the combination of melatonin (1 mmol/L) significantly enhanced the inhibitory effect of dabrafenib on cell viability (Figure 1B). By contrast, thyroid cancer cells with wild‐type BRAF were less sensitive to dabrafenib treatment, and their viability was significantly inhibited only at higher drug concentrations. CI values at different levels of growth inhibition (Fa) were also calculated using CompuSyn software. As shown in Figure S1, CI < 1 was observed in SW1736, KHM‐5M and OCUT1 cells with BRAFV600E mutant. In CAL‐62 cells of BRAFWT, CI < 1 was observed only at a high Fa level. Conclusively, melatonin combined with dabrafenib has a synergistic inhibitory effect on the viability of ATC cells.

We then assessed the effect of combination treatment on the colony formation abilities of ATC cells. As shown in Figure 1C and D, compared to single agents, the combination of melatonin and dabrafenib significantly increased the inhibition of colony formation in SW1736 and OCUT1 cells.

In addition, we next explored the potential molecular mechanisms by which combination of melatonin and dabrafenib synergistically inhibited the proliferation of ATC cells. The results showed that the expression of phosphorylated AKT protein involved in proliferation was significantly reduced after treatment with melatonin and dabrafenib in SW1736 and OCUT1 cells (Figure 1E). Taken together, these data indicated that the combination of melatonin and dabrafenib has a synergistic effect in inhibiting thyroid cancer cells proliferation by targeting AKT signalling.

### 3.2 Combination of melatonin and dabrafenib synergistically induced cell cycle arrest

**FIGURE 2:**
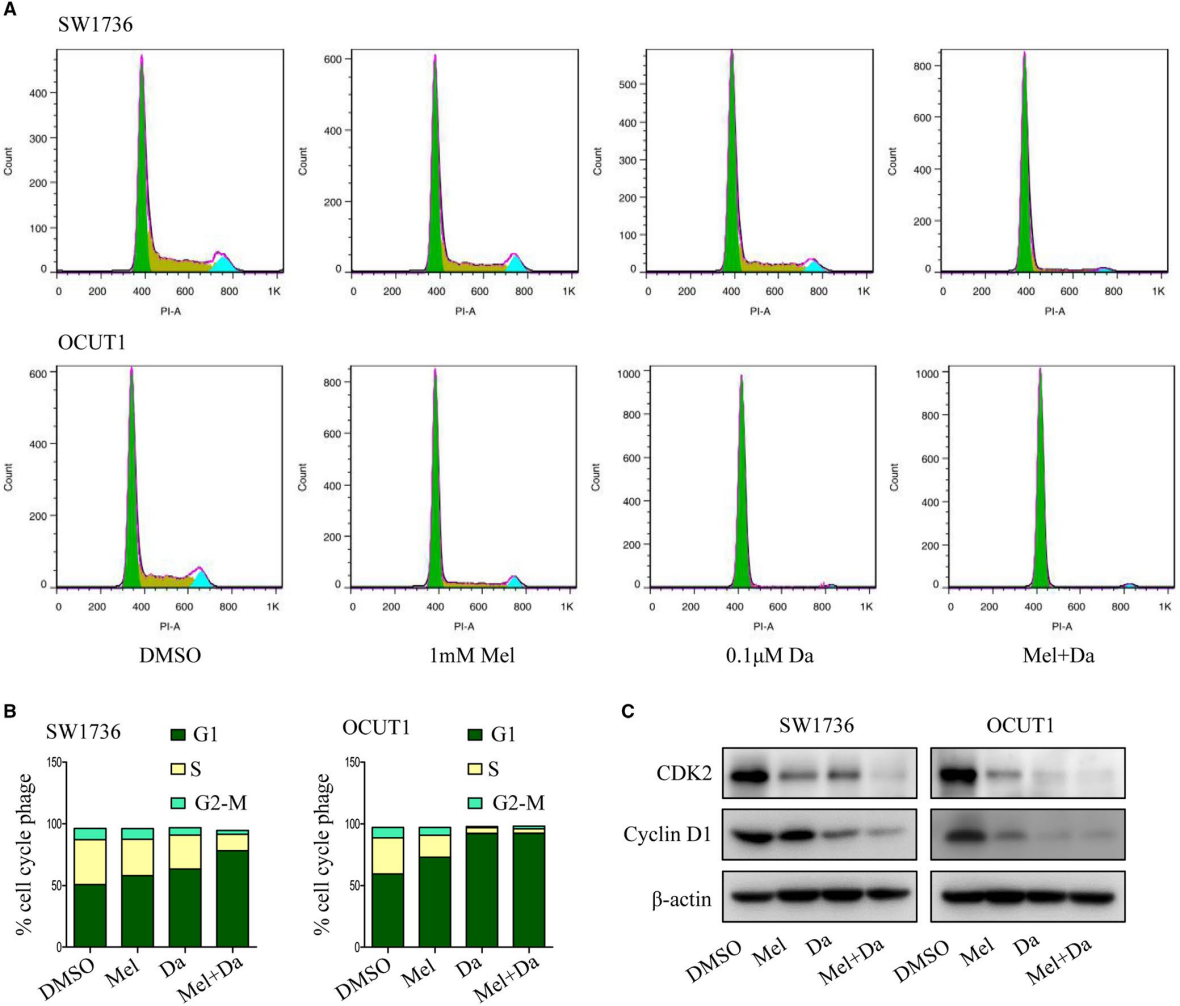
Melatonin potentiated dabrafenib‐mediated cell cycle arrest. A and B, DNA content–based cell cycle analysis was carried out in anaplastic thyroid cancer cells treated with Mel or Da alone or their combination for 48 h. The percentage of cells at each phase of the cell cycle was also quantified. C, Western blot analysis of the expression of G1/S checkpoint proteins CDK2 and cyclin D1 in SW1736 and OCUT1 cells with indicated treatment

To evaluate whether the synergistic inhibition of melatonin and dabrafenib on ATC cells growth was related to cell cycle arrest, SW1736 and OCUT1 cells were treated with melatonin or dabrafenib alone or together for 48 hours, followed by cell cycle analysis. As shown in Figure 2A and B, the combination of melatonin (1 mmol/L) and dabrafenib (0.1 μmol/L) significantly increased the number of cells in the G1 phase compared to monotherapy. Moreover, we also examined the expression levels of several key proteins involved in G1/S cell cycle regulation, and the results showed that co‐treatment with melatonin and dabrafenib significantly inhibited the expression of CDK2 and cyclin D1 (Figure 2C). These results suggested that the combination treatment of melatonin and dabrafenib synergistically induced cell cycle arrest in G1 phase.

### 3.3 Melatonin synergized with dabrafenib to induce apoptosis in anaplastic thyroid cancer cells

**FIGURE 3:**
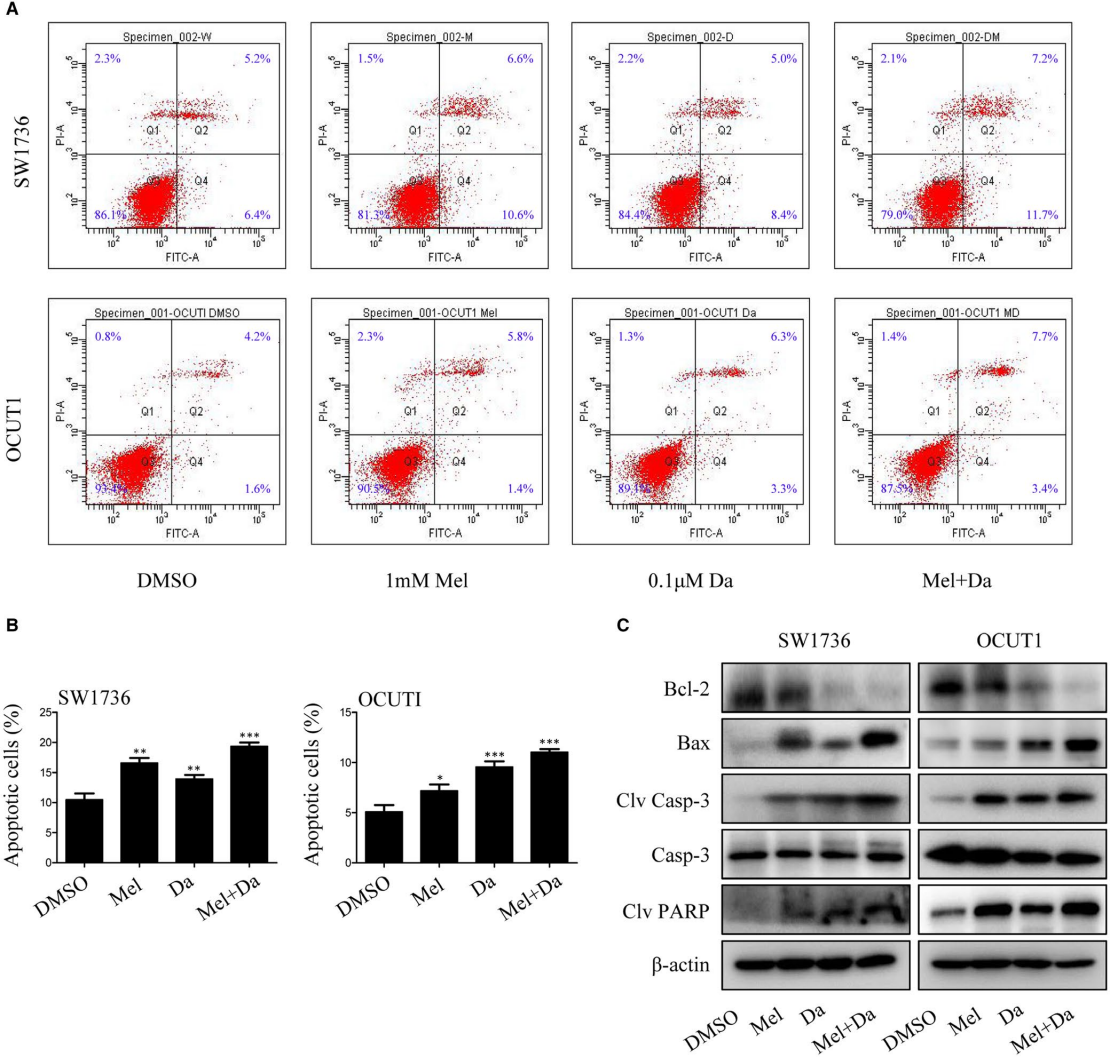
Melatonin increased dabrafenib‐induced apoptosis by activating caspase signalling pathway. SW1736 and OCUT1 cells were treated with Mel or Da alone or their combination for 48 h. A and B, The apoptosis was determined by FACS analysis, and the percentage of apoptotic cells was calculated. C, Western blot analysis of the expression of Bcl‐2, Bax, cleaved caspase‐3, caspase‐3 and cleaved PARP proteins. Data were presented as mean ± SD of three independent experiments. The level of significance was indicated by ***P < .001, **P < .01, *P < .05

To further determine whether the synergistic inhibition of melatonin and dabrafenib on the growth of ATC cells was related to the increased activation of apoptotic pathway, we then analysed the effects of melatonin and dabrafenib as single agents or in combination on the apoptosis of cancer cells through FACS analysis. As shown in Figure 3A and B, compared to monotherapy, the combination of melatonin (1 mmol/L) and dabrafenib (0.1 μmol/L) significantly increased the apoptosis of SW1736 and OCUT1 cells, leading to more apoptotic cell populations.

Considering that apoptosis induction usually occurs following a series of apoptosis‐related molecular events, we then examined the expression changes of certain apoptosis‐related proteins in SW1736 and OCUT1 cells treated with melatonin and dabrafenib. As shown in Figure 3C, compared with melatonin or dabrafenib alone, co‐treatment with these two agents resulted in decreased expression of anti‐apoptotic protein Bcl‐2, largely increased the expression of pro‐apoptotic protein Bax and also up‐regulated the cleavage of pro‐apoptotic proteins caspase‐3 and PARP, confirming that the combination of melatonin and dabrafenib indeed induced more tumour cell apoptosis and also suggesting thyroid cancer cell growth inhibition caused by melatonin and dabrafenib was achieved at least partially by promoting apoptosis.

### 3.4 Melatonin and dabrafenib had synergistic inhibitory effects on the migration and invasion of anaplastic thyroid cancer cells

**FIGURE 4:**
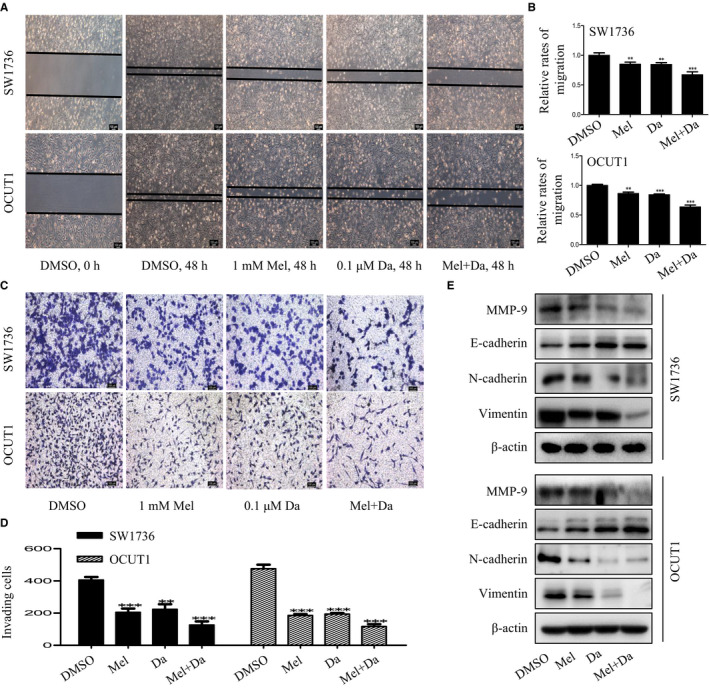
Melatonin enhanced the inhibitory effect of dabrafenib on cell migration and invasion. A and B, Scratch assays of SW1736 and OCUT1 cells after treated with Mel or Da alone or their combination for 48 h. Representative images were shown (A), and the relative rates of migration were calculated (B). C and D, Transwell invasion assays of SW1736 and OCUT1 cells after treated with Mel or Da alone or their combination for 48 h. Representative images were shown (C), and the number of invading cells was calculated (D). E, The expression of EMT markers MMP‐9, E‐cadherin, N‐cadherin and vimentin was, respectively, detected in anaplastic thyroid cancer cells with indicated treatment by Western blot assays. Data were presented as mean ± SD of three independent experiments. The level of significance was indicated by ***P < .001, **P < .01

We also evaluated the synergistic regulation of melatonin and dabrafenib on the migration and invasion abilities of ATC cells by performing scratch assays and transwell invasion assays, respectively. As shown in Figure 4A and B, monotherapy with melatonin or dabrafenib alone inhibited cell migration, but the combination of these two agents significantly enhanced the inhibitory effect of cell migration. Consistent with the inhibition of cell migration, the combinational use of melatonin and dabrafenib also led to a significant reduction in the invasiveness of ATC cells compared to single agents (Figure 4C and D).

As epithelial‐mesenchymal transition (EMT) was a vital step in the process of tumour migration, invasion and metastasis,^30^ we then observed the synergistic effect of melatonin and dabrafenib as single agents or together on the expression of several key proteins involved in the EMT process of ATC cells. As shown in Figure 4E, the combination of melatonin and dabrafenib led to a significant decreased expression of EMT inducer (MMP‐9) and mesenchymal markers (N‐cadherin and vimentin), but up‐regulated the expression of epithelial markers (E‐cadherin). These results together demonstrated the synergistic inhibitory effects of melatonin and dabrafenib on the migration and invasion of ATC cells.

### 3.5 Melatonin synergistically enhanced dabrafenib‐induced growth inhibition of anaplastic thyroid cancer cells by down‐regulating hTERT signalling

**FIGURE 5:**
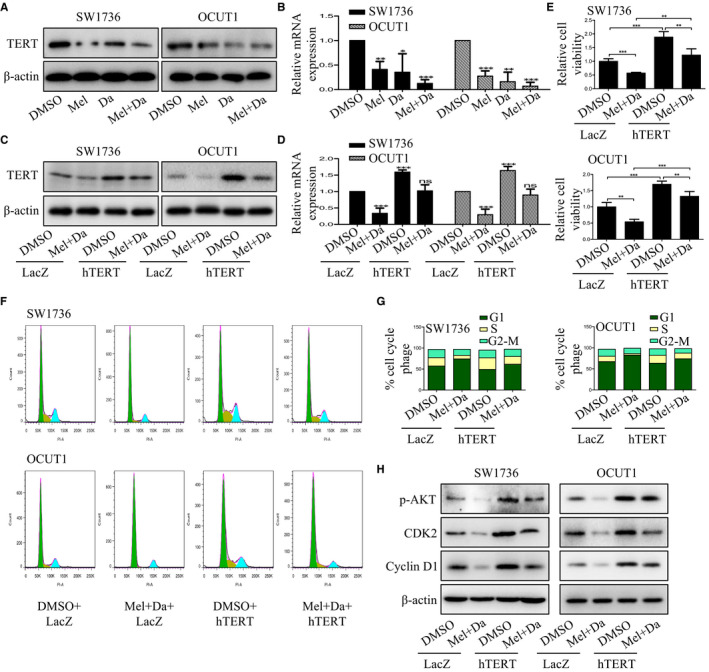
Combination of melatonin and dabrafenib inhibited the growth of anaplastic thyroid cancer cells by down‐regulating hTERT expression. A and B, SW1736 and OCUT1 cells were treated with Mel (1 mmol/L) or Da (0.1 μmol/L) alone or their combination. After 48 h, the expression of hTERT was analysed by Western blotting (A) and qPCR (B), respectively. C and D, SW1736 and OCUT1 cells were pre‐treated with Mel (1 mmol/L) and Da (0.1 μmol/L) for 24 h, and then treated with hTERT overexpression or lac Z control plasmids. After 48 h, the expression of hTERT was analysed by Western blotting (C) and qPCR (D), respectively. E, CCK‐8 assay was carried out in anaplastic thyroid cancer cells transfected with hTERT overexpression or Lac Z control plasmids after treated with Mel (1 mmol/L) in combination with Da (0.1 μmol/L). F and G, Cell cycle assay was performed in SW1736 and OCUT1 cells with indicated treatment, and the percentage of cells at each phase of cell cycle was also quantified. H, SW1736 and OCUT1 cells were pre‐treated with Mel (1 mmol/L) and Da (0.1 μmol/L) for 24 h, and then treated with hTERT overexpression or lac Z control plasmids. After 48 h, the phosphorylation of AKT and the expression of CDK2 and cyclin D1 were detected by Western blotting

We next explored the molecular events that may be involved in the growth inhibition of ATC cells mediated by co‐treatment with melatonin and dabrafenib. hTERT, a major component of telomerase, has been shown to contribute to transforming normal human cells into cancer cells. Studies have suggested that inhibition of hTERT significantly reduced the proliferation, invasion and migration abilities of ATC cells.^31^ To determine whether combination treatment with melatonin and dabrafenib could affect hTERT signalling in ATC cells, we treated melatonin or dabrafenib alone or together, and examined the expression of hTERT. We found that co‐treatment with melatonin and dabrafenib significantly reduced the expression of hTERT at protein and mRNA levels (Figure 5A and B).

To further identify hTERT expression was involved in the synergistic growth inhibition of melatonin and dabrafenib in ATC cells, SW1736 and OCUT1 cells were transfected with hTERT control vector or overexpression plasmids after pre‐treatment with melatonin and dabrafenib. After 48 hours of treatment, the expression of hTERT protein, cell viability and cell cycle arrest was determined. The results showed that the expression of hTERT was significantly increased in both cell lines (Figure 5C and D). In addition, the decreased cell viability caused by the combination of melatonin and dabrafenib was reversed by hTERT overexpression (Figure 5E). Similarly, overexpression of hTERT significantly inhibited the induction of cell cycle arrest at G1‐phase mediated by combination treatment (Figure 5F and G). Overexpression of hTERT also increased the phosphorylation of AKT and significantly promoted the expression of CDK2 and cyclin D1. These results showed that the enhanced inhibitory function of melatonin and dabrafenib on ATC cells growth is at least partially achieved by inhibiting hTERT signalling pathway.

## 4 DISCUSSION

In the past few years, most thyroid cancer treatments have targeted known oncogenic mutations, such as BRAFV600E, to convert the progression of thyroid cancer. In view of the high frequency of BRAFV600E mutations in ATC, the use of selective BRAFV600E inhibitors was a good choice for improving therapeutic specificity and reducing toxicity. However, while the initial response rate was impressive, the persistence of the response was limited by the development of drug resistance.^32^, ^33^ In this study, we demonstrated that the combination of melatonin and BRAF‐targeting agent dabrafenib exhibited synergistic anticancer effects in ATC cells, as characterized by increased cell viability inhibition, colony formation inhibition, migration and invasion inhibition, cell cycle arrest, and apoptosis induction. In addition, we also demonstrated that the increased inhibitory effect of melatonin and dabrafenib on hTERT expression in ATC cells, elucidating the possible molecular mechanisms involved in the anti‐tumour effects of this combination therapy.

Cell apoptosis is an important manifestation of cell death, which is closely related to the development of tumours.^34^ The caspase‐dependent apoptotic signals are key regulators in this process, which is activated by mitochondria releasing cytochrome‐c, thereby activating downstream caspase‐3 and the degradation of certain important cellular proteins, such as PARP.^35^ In our study, we functionally investigated the synergistic regulation of melatonin and dabrafenib on apoptosis induction of ATC cells. Although dabrafenib itself caused significant apoptosis of tumour cells, such apoptosis induction was greatly enhanced by its combination with melatonin. Moreover, the combination of melatonin and dabrafenib resulted in an increased activation of the caspase‐dependent apoptotic signalling pathway, as evidenced by increasing the expression of Bax, decreasing the expression of Bcl‐2 and up‐regulating the expression of cleaved caspase‐3 and PARP. Taken together, these results suggested that the enhanced anti‐tumour effect of melatonin and dabrafenib was partly based on the increased activation of the caspase‐dependent apoptotic pathway, which was consistent with the previous studies that melatonin alone or combined with chemotherapeutic agents induced apoptosis in several cancers.^36^, ^37^

The main cause of death in ATC patients is related to its profound metastasis and invasion characteristics^38^ EMT, a process in which epithelial cells transform to mesenchymal phenotype and subsequently become highly mobile, has been shown to affect the progression of cancer cell migration and invasion. The acquisition of mesenchymal markers, such as N‐cadherin and vimentin, and the absence of epithelial markers, such as E‐cadherin, are the hallmarks of EMT. Our results in this study demonstrated that compared with the use of melatonin or dabrafenib alone, the combination of these two agents significantly increased the expression of E‐cadherin and decreased the expression of N‐cadherin and vimentin, thus inhibiting the EMT process, implying that the enhanced inhibition of cell growth mediated by melatonin and dabrafenib was related to the increased inhibition of cell metastasis and invasion of ATC cells.

hTERT is a major component of human telomerase that prolongs the end of chromosomes and maintains chromosomal stability, resulting in cellular immortalization and tumorigenesis.^39^ For a long time, studies on hTERT were mainly focused on its ability to maintain telomere length to continuously promote cell proliferation. However, in recent years, hTERT has also been found to have non‐telomere‐dependent functions,^40^ such as regulation of gene expression, cellular signalling pathways, cell cycle, protection of mitochondrial DNA and regulation of DNA damage response.^41^, ^42^, ^43^, ^44^, ^45^ It was usually overexpressed in a variety of tumours, including thyroid cancer.^31^ Accumulating evidence has suggested that overexpression of hTERT was not only related to the aggressive behaviours of thyroid cancer cells,^46^ but also predicted the early recurrence of thyroid cancer patients.^47^ Knockdown of hTERT resulted in decreased proliferation, invasion and migration abilities of ATC cells.^31^ Moreover, hTERT was found to induce thyroid cancer cell proliferation by regulating PTEN/AKT signalling pathway.^48^ Considering that melatonin exerted its anti‐tumour effects by inhibiting the expression of hTERT and the activity of telomerase,^49^ we deduced that melatonin might potentiate dabrafenib‐mediated growth inhibition of ATC cells in part by inhibiting hTERT signalling. Our results showed that the combination of melatonin and dabrafenib significantly reduced the expression of hTERT at mRNA and protein levels. Notably, overexpression of hTERT rescued the inhibition of cell viability caused by co‐treatment with melatonin and dabrafenib. Furthermore, the induction of cell cycle arrest at G1 phase caused by such combinational treatment was significantly attenuated when hTERT was overexpressed. Collectively, all of these results indicated that melatonin synergized with dabrafenib to induce growth inhibition in ATC cells partly through inhibiting hTERT signalling, and such signalling inhibition may be a potential therapeutic target in thyroid cancer. However, further studies are needed to elucidate the molecular mechanisms by which melatonin and dabrafenib synergistically inhibited hTERT expression. Besides, melatonin and dabrafenib should be further tested in vivo to evaluate their antineoplastic effect in nude mice model.

## 5 CONCLUSIONS

**FIGURE 6:**
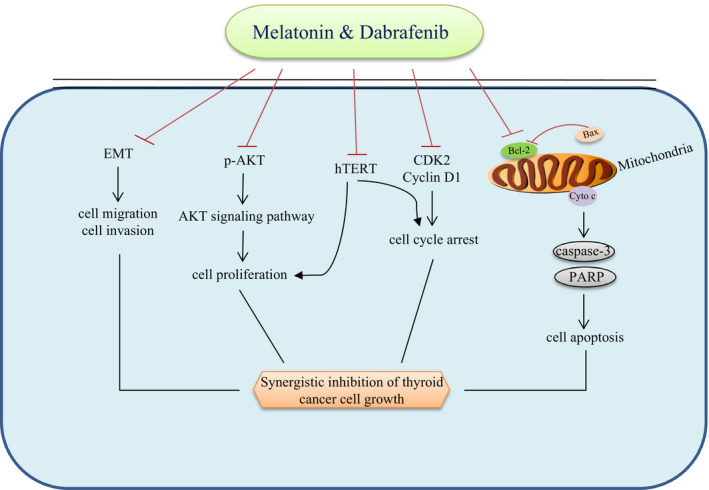
The schematic diagram of the molecular mechanisms by which melatonin synergized dabrafenib to regulate the growth of anaplastic thyroid cancer cells. The symbol (⊦) indicates negative regulation. The arrow (→) indicates positive regulation

In summary, our results suggested that melatonin and dabrafenib synergistically inhibited the growth of anaplastic thyroid cancer cells, as evidenced by synergistic inhibition of proliferation, migration and invasion, and synergistic enhancement of cell cycle arrest and apoptosis induction. In addition, we also investigated the molecular mechanisms that may be involved in this combination therapy, and found that melatonin synergized the anti‐tumour effect of dabrafenib by inhibiting hTERT signalling in human anaplastic thyroid cancer cells (Figure 6). All these data indicated that the combinational use of melatonin and BRAF‐targeting agent dabrafenib has the potential to become a novel approach in the treatment of anaplastic thyroid cancer.

## CONFLICT OF INTEREST

The authors confirm that there are no conflicts of interest.

## AUTHOR CONTRIBUTIONS

**Yina Liao:** Data curation (equal); Formal analysis (equal); Investigation (equal); Writing‐original draft (lead). **Yao Gao:** Data curation (equal); Formal analysis (equal); Investigation (equal). **An Chang:** Conceptualization (equal); Formal analysis (equal); Investigation (equal). **Zongjuan Li:** Investigation (supporting). **Huayu Wang:** Formal analysis (equal); Investigation (equal); Resources (supporting). **Jing Cao:** Data curation (equal); Formal analysis (equal); Funding acquisition (supporting); Resources (equal); Supervision (supporting). **Wei Gu:** Data curation (equal); Investigation (equal); Supervision (equal); Writing‐original draft (equal). **Ranran Tang:** Data curation (equal); Formal analysis (equal); Funding acquisition (equal); Investigation (equal); Methodology (equal); Project administration (equal); Writing‐original draft (equal); Writing‐review & editing (equal).

## Supporting information

Figure S1

## Data Availability

The data used to support the findings of this study are available from the corresponding author upon request.

## References

[1] Lim H , Devesa SS , Sosa JA , Check D , Kitahara CM . Trends in thyroid cancer incidence and mortality in the United States, 1974‐2013. JAMA. 2017;317:1338‐1348. 28362912 10.1001/jama.2017.2719 PMC8216772

[2] Siegel RL , Miller KD , Jemal A . Cancer statistics, 2018. CA Cancer J Clin. 2018;68:7‐30. 29313949 10.3322/caac.21442

[3] Bray F , Ferlay J , Soerjomataram I , Siegel RL , Torre LA , Jemal A . Global cancer statistics 2018: GLOBOCAN estimates of incidence and mortality worldwide for 36 cancers in 185 countries. CA Cancer J Clin. 2018;68:394‐424. 30207593 10.3322/caac.21492

[4] Tuttle RM , Ball DW , Byrd D , et al. Thyroid carcinoma. J Natl Compr Canc Netw. 2010;8:1228‐1274. 21081783 10.6004/jnccn.2010.0093

[5] Hadoux J , Pacini F , Tuttle RM , Schlumberger M . Management of advanced medullary thyroid cancer. Lancet Diabetes Endocrinol. 2016;4:64‐71. 26608066 10.1016/S2213-8587(15)00337-X

[6] Keutgen XM , Sadowski SM , Kebebew E . Management of anaplastic thyroid cancer. Gland Surg. 2015;4:44‐51. 25713779 10.3978/j.issn.2227-684X.2014.12.02 PMC4321056

[7] Ferrari SM , Elia G , Ragusa F , et al. Novel treatments for anaplastic thyroid carcinoma. Gland Surg. 2020;9:S28‐S42. 32055496 10.21037/gs.2019.10.18 PMC6995904

[8] Saini S , Tulla K , Maker AV , Burman KD , Prabhakar BS . Therapeutic advances in anaplastic thyroid cancer: a current perspective. Mol Cancer. 2018;17:154. 30352606 10.1186/s12943-018-0903-0 PMC6198524

[9] Smallridge RC , Ain KB , Asa SL , et al. American Thyroid Association guidelines for management of patients with anaplastic thyroid cancer. Thyroid. 2012;22:1104‐1139. 23130564 10.1089/thy.2012.0302

[10] Lim AM , Taylor GR , Fellowes A , et al. BRAF inhibition in BRAFV600E‐positive anaplastic thyroid carcinoma. J Natl Compr Canc Netw. 2016;14:249‐254. 26957611 10.6004/jnccn.2016.0030

[11] Cavalheiro BG , Matos LL , Leite AK , Kulcsar MA , Cernea CR , Brandao LG . Surgical treatment for thyroid carcinoma: retrospective study with 811 patients in a Brazilian tertiary hospital. Arch Endocrinol Metab. 2016;60:472‐478. 27737324 10.1590/2359-3997000000209 PMC10118633

[12] Woodward EL , Biloglav A , Ravi N , et al. Genomic complexity and targeted genes in anaplastic thyroid cancer cell lines. Endocr Relat Cancer. 2017;24:X2. 28673895 10.1530/ERC-16-0522e

[13] Santarpia L , El‐Naggar AK , Cote GJ , Myers JN , Sherman SI . Phosphatidylinositol 3‐kinase/akt and ras/raf‐mitogen‐activated protein kinase pathway mutations in anaplastic thyroid cancer. J Clin Endocrinol Metab. 2008;93:278‐284. 17989125 10.1210/jc.2007-1076

[14] Torii S , Yamamoto T , Tsuchiya Y , Nishida E . ERK MAP kinase in G cell cycle progression and cancer. Cancer Sci. 2006;97:697‐702. 16800820 10.1111/j.1349-7006.2006.00244.x PMC11158792

[15] Falchook GS , Millward M , Hong D , et al. BRAF inhibitor dabrafenib in patients with metastatic BRAF‐mutant thyroid cancer. Thyroid. 2015;25:71‐77. 25285888 10.1089/thy.2014.0123 PMC4291160

[16] Ljubas J , Ovesen T , Rusan M . A systematic review of phase II targeted therapy clinical trials in anaplastic thyroid cancer. Cancers. 2019;11:943. 10.3390/cancers11070943 PMC6678800 31277524

[17] Subbiah V , Kreitman RJ , Wainberg ZA , et al. Dabrafenib and trametinib treatment in patients with locally advanced or metastatic BRAF V600‐mutant anaplastic thyroid cancer. J Clin Oncol. 2018;36:7‐13. 29072975 10.1200/JCO.2017.73.6785 PMC5791845

[18] Cabanillas ME , Patel A , Danysh BP , Dadu R , Kopetz S , Falchook G . BRAF inhibitors: experience in thyroid cancer and general review of toxicity. Hormones Cancer. 2015;6:21‐36. 25467940 10.1007/s12672-014-0207-9 PMC4312215

[19] Liu T , Jin L , Chen M , et al. Ku80 promotes melanoma growth and regulates antitumor effect of melatonin by targeting HIF1‐α dependent PDK‐1 signaling pathway. Redox Biol. 2019;25:101197. 31023624 10.1016/j.redox.2019.101197 PMC6859552

[20] Reiter RJ , Mayo JC , Tan DX , Sainz RM , Alatorre‐Jimenez M , Qin L . Melatonin as an antioxidant: under promises but over delivers. J Pineal Res. 2016;61:253‐278. 27500468 10.1111/jpi.12360

[21] Goradel NH , Asghari MH , Moloudizargari M , Negahdari B , Haghi‐Aminjan H , Abdollahi M . Melatonin as an angiogenesis inhibitor to combat cancer: mechanistic evidence. Toxicol Appl Pharmacol. 2017;335:56‐63. 28974455 10.1016/j.taap.2017.09.022

[22] Mauriz JL , Collado PS , Veneroso C , Reiter RJ , González‐Gallego J . A review of the molecular aspects of melatonin's anti‐inflammatory actions: recent insights and new perspectives. J Pineal Res. 2013;54:1‐14. 22725668 10.1111/j.1600-079X.2012.01014.x

[23] Calvo JR , González‐Yanes C , Maldonado MD . The role of melatonin in the cells of the innate immunity: a review. J Pineal Res. 2013;55:103‐120. 23889107 10.1111/jpi.12075

[24] Cabrera J , Negrín G , Estévez F , Loro J , Reiter RJ , Quintana J . Melatonin decreases cell proliferation and induces melanogenesis in human melanoma SK‐MEL‐1 cells. J Pineal Res. 2010;49:45‐54. 20459460 10.1111/j.1600-079X.2010.00765.x

[25] Alonso‐González C , González A , Martínez‐Campa C , et al. Melatonin enhancement of the radiosensitivity of human breast cancer cells is associated with the modulation of proteins involved in estrogen biosynthesis. Cancer Lett. 2016;370:145‐152. 26497762 10.1016/j.canlet.2015.10.015

[26] Fan C , Pan Y , Yang Y , et al. HDAC1 inhibition by melatonin leads to suppression of lung adenocarcinoma cells via induction of oxidative stress and activation of apoptotic pathways. J Pineal Res. 2015;59:321‐333. 26184924 10.1111/jpi.12261

[27] Zou ZW , Liu T , Li Y , et al. Melatonin suppresses thyroid cancer growth and overcomes radioresistance via inhibition of p65 phosphorylation and induction of ROS. Redox Biol. 2018;16:226‐236. 29525603 10.1016/j.redox.2018.02.025 PMC5854931

[28] Hao J , Fan W , Li Y , et al. Melatonin synergizes BRAF‐targeting agent vemurafenib in melanoma treatment by inhibiting iNOS/hTERT signaling and cancer‐stem cell traits. J Exp Clin Cancer Res. 2019;38:48. 30717768 10.1186/s13046-019-1036-z PMC6360719

[29] Gao Y , Xiao X , Zhang C , et al. Melatonin synergizes the chemotherapeutic effect of 5‐fluorouracil in colon cancer by suppressing PI3K/AKT and NF‐κB/iNOS signaling pathways. J Pineal Res. 2017;62:e12380. 10.1111/jpi.12380 27865009

[30] Ban Z , He J , Tang Z , Zhang L , Xu Z . LRG‐1 enhances the migration of thyroid carcinoma cells through promotion of the epithelial‐mesenchymal transition by activating MAPK/p38 signaling. Oncol Rep. 2019;41:3270‐3280. 31002347 10.3892/or.2019.7123 PMC6488982

[31] Maggisano V , Celano M , Lombardo GE , et al. Silencing of hTERT blocks growth and migration of anaplastic thyroid cancer cells. Mol Cell Endocrinol. 2017;448:34‐40. 28288903 10.1016/j.mce.2017.03.007

[32] Iyer PC , Dadu R , Ferrarotto R , et al. Real‐world experience with targeted therapy for the treatment of anaplastic thyroid carcinoma. Thyroid. 2018;28:79‐87. 29161986 10.1089/thy.2017.0285 PMC6425981

[33] Karoulia Z , Gavathiotis E , Poulikakos PI . New perspectives for targeting RAF kinase in human cancer. Nat Rev Cancer. 2017;17:676‐691. 28984291 10.1038/nrc.2017.79 PMC6000833

[34] Jia L , Zhu Z , Li H , Li Y . Shikonin inhibits proliferation, migration, invasion and promotes apoptosis in NCI‐N87 cells via inhibition of PI3K/AKT signal pathway. Artif Cells Nanomed Biotechnol. 2019;47:2662‐2669. 31257936 10.1080/21691401.2019.1632870

[35] Yi C , Zhang Y , Yu Z , et al. Melatonin enhances the anti‐tumor effect of fisetin by inhibiting COX‐2/iNOS and NF‐κB/p300 signaling pathways. PLoS One. 2014;9:e99943. 25000190 10.1371/journal.pone.0099943 PMC4085069

[36] Martín‐Renedo J , Mauriz JL , Jorquera F , Ruiz‐Andrés O , González P , González‐Gallego J . Melatonin induces cell cycle arrest and apoptosis in hepatocarcinoma HepG2 cell line. J Pineal Res. 2008;45:532‐540. 19012662 10.1111/j.1600-079X.2008.00641.x

[37] Kim JH , Jeong SJ , Kim B , Yun SM , Choi DY , Kim SH . Melatonin synergistically enhances cisplatin‐induced apoptosis via the dephosphorylation of ERK/p90 ribosomal S6 kinase/heat shock protein 27 in SK‐OV‐3 cells. J Pineal Res. 2012;52:244‐252. 22050627 10.1111/j.1600-079X.2011.00935.x

[38] Guo Z , Hardin H , Lloyd RV . Cancer stem‐like cells and thyroid cancer. Endocr Relat Cancer. 2014;21:T285‐300. 24788702 10.1530/ERC-14-0002

[39] Stewart SA , Hahn WC , O'Connor BF , et al. Telomerase contributes to tumorigenesis by a telomere length‐independent mechanism. Proc Natl Acad Sci USA. 2002;99:12606‐12611. 12193655 10.1073/pnas.182407599 PMC130507

[40] Cong Y , Shay JW . Actions of human telomerase beyond telomeres. Cell Res. 2008;18:725‐732. 18574498 10.1038/cr.2008.74

[41] Sharma GG , Gupta A , Wang H , et al. hTERT associates with human telomeres and enhances genomic stability and DNA repair. Oncogene. 2003;22:131‐146. 12527915 10.1038/sj.onc.1206063

[42] Ghosh A , Saginc G , Leow SC , et al. Telomerase directly regulates NF‐κB‐dependent transcription. Nat Cell Biol. 2012;14:1270‐1281. 23159929 10.1038/ncb2621

[43] Xiang H , Wang J , Mao Y , Liu M , Reddy VN , Li DW . Human telomerase accelerates growth of lens epithelial cells through regulation of the genes mediating RB/E2F pathway. Oncogene. 2002;21:3784‐3791. 12032846 10.1038/sj.onc.1205455

[44] Ahmed S , Passos JF , Birket MJ , et al. Telomerase does not counteract telomere shortening but protects mitochondrial function under oxidative stress. J Cell Sci. 2008;121:1046‐1053. 18334557 10.1242/jcs.019372

[45] Lee J , Sung YH , Cheong C , et al. TERT promotes cellular and organismal survival independently of telomerase activity. Oncogene. 2008;27:3754‐3760. 18223679 10.1038/sj.onc.1211037

[46] Liu R , Zhang T , Zhu G , Xing M . Regulation of mutant TERT by BRAF V600E/MAP kinase pathway through FOS/GABP in human cancer. Nat Commun. 2018;9:579. 29422527 10.1038/s41467-018-03033-1 PMC5805723

[47] Xing M , Liu R , Liu X , et al. BRAF V600E and TERT promoter mutations cooperatively identify the most aggressive papillary thyroid cancer with highest recurrence. J Clin Oncol. 2014;32:2718‐2726. 25024077 10.1200/JCO.2014.55.5094 PMC4145183

[48] Zhang H , Hu N . Telomerase reverse transcriptase induced thyroid carcinoma cell proliferation through PTEN/AKT signaling pathway. Mol Med Rep. 2018;18:1345‐1352. 29901196 10.3892/mmr.2018.9119 PMC6072153

[49] Tang YL , Sun X , Huang LB , et al. Melatonin inhibits MLL‐rearranged leukemia via RBFOX3/hTERT and NF‐κB/COX‐2 signaling pathways. Cancer Lett. 2019;443:167‐178. 30550850 10.1016/j.canlet.2018.11.037

