# Supplementary figures and images for: Melatonin synergizes BRAF‐targeting agent dabrafenib for the treatment of anaplastic thyroid cancer by inhibiting AKT/hTERT signalling
Source: J Cell Mol Med. 2020 Sep 15;24(20):12119–30. doi: 10.1111/jcmm.15854 (PMC7579709; doi:10.1111/jcmm.15854)

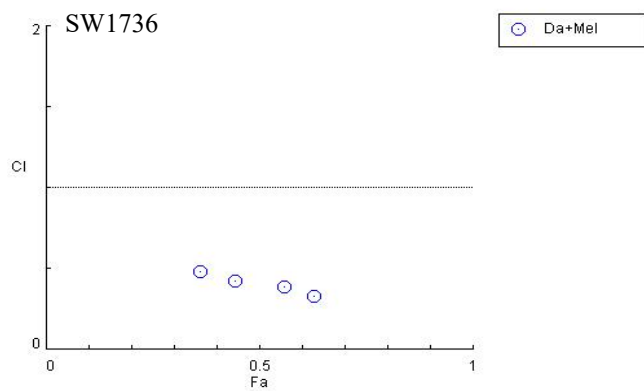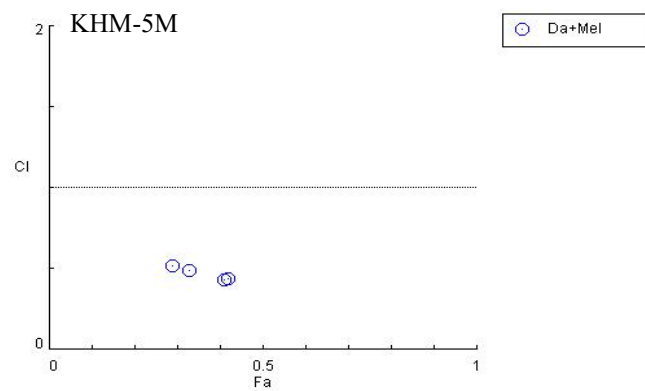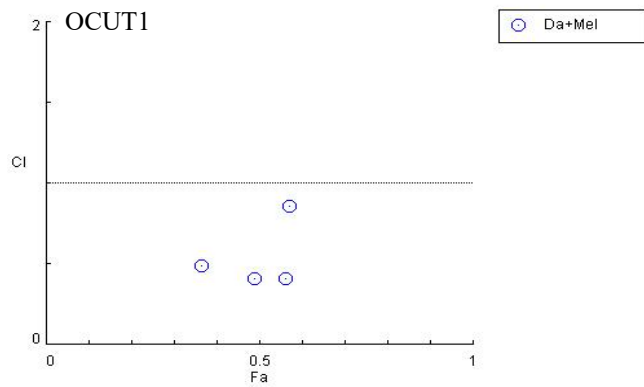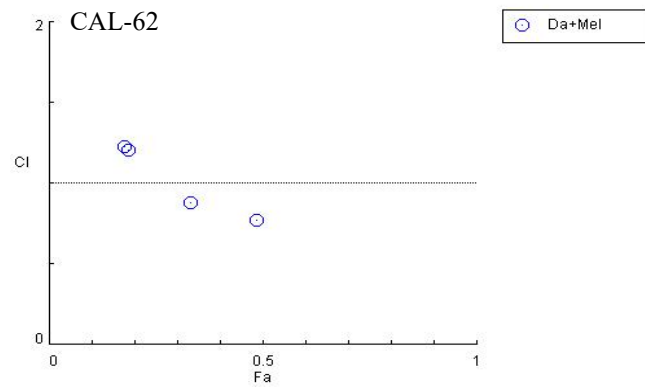

Supplement: Supplementary file 1 — Figure S1 [file JCMM-24-12119-s001.pdf]
